# Supplementary material for: Structural dynamics of the E6AP/UBE3A-E6-p53 enzyme-substrate complex
Source: Nat Commun. 2018 Oct 25;9:4441. doi: 10.1038/s41467-018-06953-0 (PMC6202321; doi:10.1038/s41467-018-06953-0)
Supplement: Supplementary file 1 — Supplementary Information [file 41467_2018_6953_MOESM1_ESM.pdf]

Structural dynamics of the E6AP/UBE3A-E6-p53 enzyme-substrate complex

Sailer *et al*.

Supplementary Information

Supplementary Fig. 1

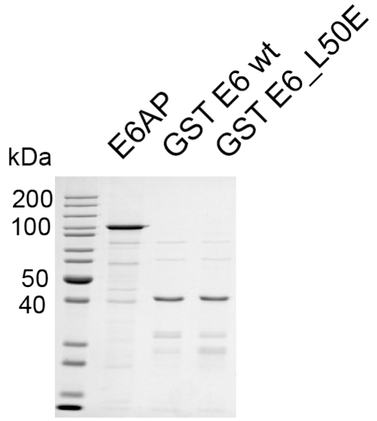

Supplementary Fig. 1 Proteins used for XL-MS experiments.

SDS-polyacrylamide gel showing preparations of E6AP, wild-type HPV-16 E6 (E6 wt), and an E6 mutant that does not bind to E6AP (E6_ L50E) used for XL-MS experiments (E6 proteins were expressed as GST fusion proteins). Proteins were visualized by Coomassie blue staining. One percent of the protein amount used for XL-MS was loaded per lane.

Supplementary Fig. 2

E6AP

```
        10         20         30         40         50
MKRAAAKHLI ERYYHQLTEG CGNEACTNEF CASCPTFLRM DNNAAAIKAL

        60         70         80         90        100
ELYKINAKLC DPHPSKKGAS SAYLENSKGA PNNSCSEIKM NKKGARIDFK

       110        120        130        140        150
DVTYLTEEKV YEILELCRER EDYSPLIRVI GRVFSSAEAL VQSFRKVKQH

       160        170        180        190        200
TKEELKSLQA KDEDKDEDEK EKAACSAAAM EEDSEASSSR IGDSSQGDNN

       210        220        230        240        250
LQKLGPDDVS VDIDAIRRVY TRLLSNEKIE TAFLNALVYL SPNVECDLTY

       260        270        280        290        300
HNVYSRDPNY LNLFIIVMEN RNLHSPEYLE MALPLFCKAM SKLPLAAQGK

       310        320        330        340        350
LIRLWSKYNA DQIRRMMETF QQLITYKVIS NEFNSRNLVN DDDAIVAASK

       360        370        380        390        400
CLKMVYYANV VGGEVDTNHN EEDDEEPIPE SSELTLQELL GEERRNKKGP

       410        420        430        440        450
RVDPLETELG VKTLDCRKPL IPFEEFINEP LNEVLEMDKD YTFFKVETEN

       460        470        480        490        500
KFSFMTCPFI LNAVTKNLGL YYDNRIRMYS ERRITVLYSL VQGQQLNPYL

       510        520        530        540        550
RLKVRRDHII DDALVRLEMI AMENPADLKK QLYVEFEGEQ GVDEGGVSKE

       560        570        580        590        600
FFQLVVEEIF NPDIGMFTYD ESTKLFWFNP SSFETEGQFT LIGIVLGLAI

       610        620        630        640        650
YNNCILDVHF PMVVYRKLMG KKGTFRDLGD SHPVLYQSLK DLLEYEGNVE

       660        670        680        690        700
DDMMITFQIS QTDLFGNPMM YDLKENGDKI PITNENRKEF VNLYSDYILN

       710        720        730        740        750
KSVEKQFKAF RRGFHMVTNE SPLKYLFRPE EIELLICGSR NLDFQALEET

       760        770        780        790        800
TEYDGGYTRD SVLIREFWEI VHSFTDEQKR LFLQFTTGTD RAPVGGLGKL

       810        820        830        840        850
KMIIAKNGPD TERLPTSHTC FNVLLLPEYS SKEKLKERLL KAITYAKGFG

852
ML
```

E6

```
        10         20         30         40         50
SFQDPQERPR KLPQLCTELQ TTIHDIILEC VYCKQQLLRR EVYDFAFRDL

        60         70         80         90        100
CIVYRDGNPY AVCDKCLKFY SKISEYRHYC YSLYGTTLEQ QYNKPLCDLL

       110        120        130        140        150
IRCINCQKPL CPEEKQRHLD KKQRFHNIRG RWTGRCMSCC RSSRTRRETQ

151
L
```

E6_L50E

```
        10         20         30         40         50
SFQDPQERPR KLPQLCTELQ TTIHDIILEC VYCKQQLLRR EVYDFAFRDE

        60         70         80         90        100
CIVYRDGNPY AVCDKCLKFY SKISEYRHYC YSLYGTTLEQ QYNKPLCDLL

       110        120        130        140        150
IRCINCQKPL CPEEKQRHLD KKQRFHNIRG RWTGRCMSCC RSSRTRRETQ

151
L
```

p53

```
        10         20         30         40         50
MEEPQSDPSV EPPLSQETFS DLWKLLPENN VLSPLPSQAM DDLMLSPDDI

        60         70         80         90        100
EQWFTEDPGP DEAPRMPEAA PPVAPAPAAP TPAAPAPAPS WPLSSSVPSQ

       110        120        130        140        150
KTYQGSYGFR LGFLHSGTAK SVTCTYSPAL NKMFCQLAKT CPVQLWVDST

       160        170        180        190        200
PPPGTRVRAM AIYKQSQHMT EVVRRCPHHE RCSDSDGLAP PQHLIRVEGN

       210        220        230        240        250
LRVEYLDDRN TFRHSVVVPY EPPEVGSDCT TIHYNYMCNS SCMGGMNRRP

       260        270        280        290        300
ILTIITLEDS SGNLLGRNSF EVRVCACPGR DRRTEEENLR KKGEPHHELP

       310        320        330        340        350
PGSTKRALPN NTSSSPQPKK KPLDGEYFTL QIRGRERFEM FRELNEALEL

       360        370        380        390 393
KDAQAGKEPG GSRAHSSHLK SKKGQSTSRH KKLMFKTEGP DSD
```

Supplementary Fig. 2 Overview of lysine residues involved in crosslinking.

All lysines are boxed in grey. Lysine residues which were identified in intra-protein crosslinks are highlighted in blue. Lysine residues which were identified in inter-protein crosslinks are highlighted in green.

Supplementary Fig. 3

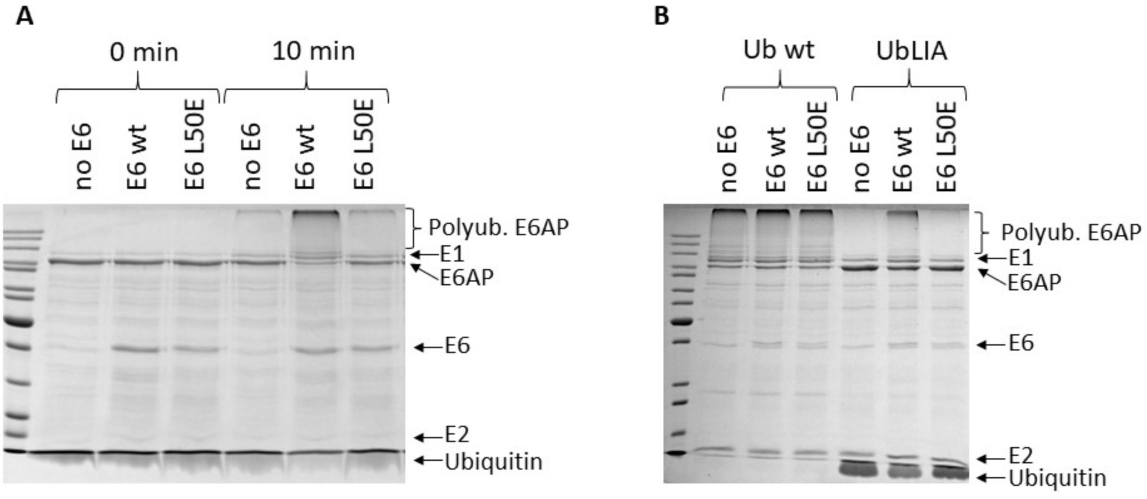

Supplementary Fig. 3 Comparison of E6AP auto-ubiquitylation in the presence of wild-type HPV-16 E6 or the E6_L50E mutant. (**A**) The E6_L50E mutant does not stimulate E6AP auto-ubiquitylation, in contrast to wild-type E6. Reactions were performed as described in Methods and stopped after 10 min. (**B**) The E6_L50E mutant does not rescue E6AP auto-ubiquitylation in the presence of the hydrophobic patch mutant UbLIA [37]. The running positions of E1, E6AP, GST-E6, E2, ubiquitin, and poly-ubiquitylated forms of E6AP are indicated.

Supplementary Fig. 4

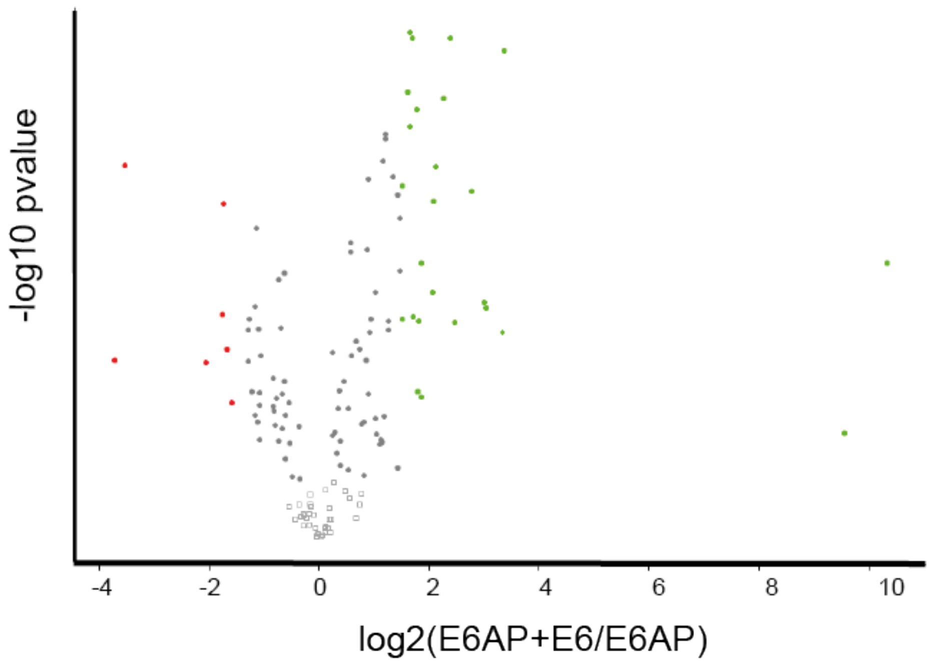

Supplementary Fig. 4. Volcano plots of quantified crosslinking sites of E6AP in the absence and presence of E6.

Shown are unique crosslinking sites (uxIDs) with an Id-Score >25. Significant enrichment was determined with respect to crosslinks identified in the E6AP sample in the absence of E6. Plotted are log2 ratio change versus the negative logarithmized p-values. Log2 = 0 indicates no enrichment, green dots indicate significant enrichment upon binding of E6 (log2ratio ≥ 1.5; p value ≤ 0.01 (two sided t-test)) and red dots a relative decrease upon binding of E6 (log2ratio ≤ -1.5; p value ≤ 0.01(two sided t-test)), respectively.

Supplementary Fig. 5

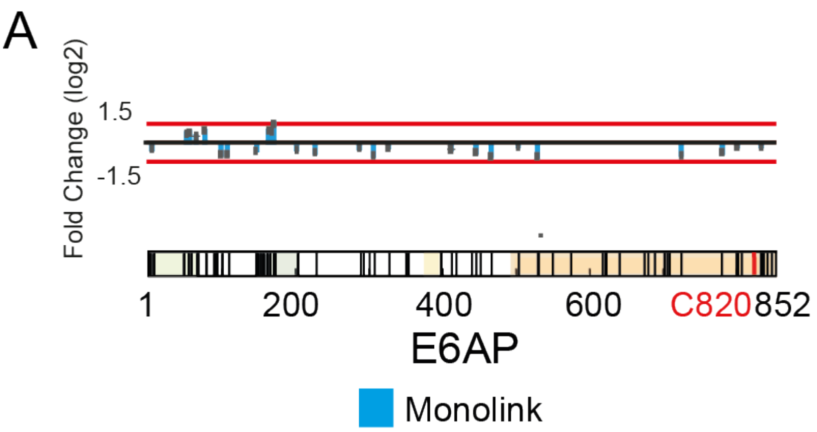

Supplementary Fig. 5 Structural dynamics of E6AP in the absence and presence of the E6_L50E mutant.

Quantification of the change in abundance of identified crosslinks within E6AP, when incubated with the binding-deficient E6_L50E mutant *vs.* E6AP alone, shows no significantly up- or down regulated monolinks and reveals overall a similar fold change pattern for monolinks as for wild-type E6, even though the significantly changed monolinks in the central region are absent in the mutant E6_L50E dataset, consistent with the notion that decreased monolinking within this region is caused by E6 binding. As monolinks are both up and down-regulated throughout the N-terminal region in the E6_L50E mutant, it is difficult to conclude with confidence what is causing the cluster of slightly positively regulated monolinks between amino acids ~70-130 in the wild-type dataset, but the basically complete absence of significantly up- or down-regulated intralinks, strongly indicates that the identified changes in the E6AP intralink pattern, as observed in the experiment to Figure 1, are caused by binding of E6 (n=2, p-value ≤ 0.05 (two sided t-test)).

Supplementary Fig. 6

A

	Light GST	Light + Heavy GST
Light-Light Links	84	35
Light-Heavy Links	0	6
Heavy-Heavy Links	0	18

B

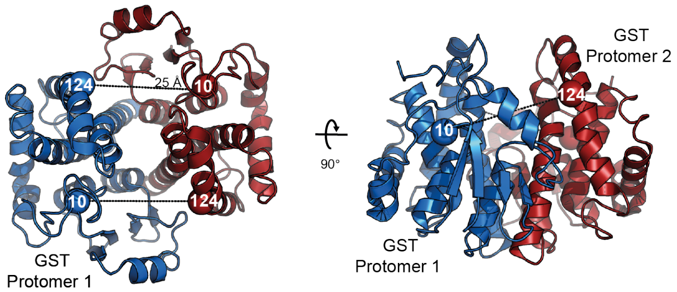

C

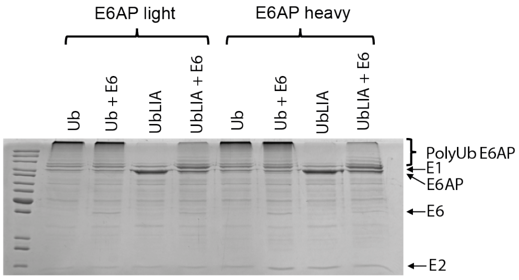

D

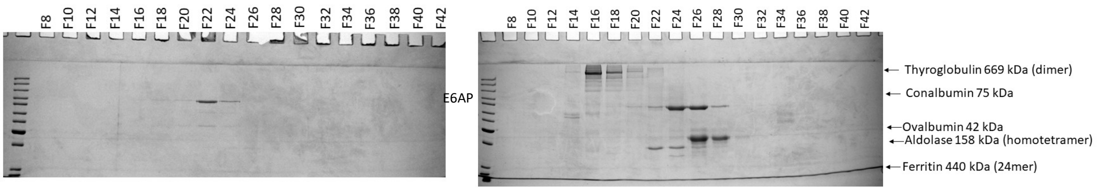

Supplementary Fig. 6 Validation of SILAC-XL-MS using dimeric GST.

(**A**) Numbers of linked peptides identified are shown after cross-linking either light GST dimers, or dimers formed following mixing of light and heavy labelled GST. Both samples were crosslinked in triplicate. The absence of either light-heavy or heavy-heavy links in the light GST sample indicates that heavy peptides were reliably assigned. (**B**) Cartoon representation of dimeric GST from *Schistosoma japonicum* (PDB: 1Y6E) showing the location of the highest scoring light-heavy interlink (black dotted line) between lysines 10 and 124 at the dimeric GST interface. (**C**) Isotope-labeling of E6AP has no influence on its ubiquitin ligase activity. Comparison of unlabeled E6AP (light) and isotope-labeled E6AP (heavy) shows no difference in their capacity for auto-ubiquitylation. Running positions of E1, E6AP, E6, E2, and of poly-ubiquitylated forms of E6AP are indicated. (**D**) E6AP appears as a monomer on SEC. Size-exclusion chromatography (Superdex 10/300 Increase column) confirms that under the conditions used (300 mM NaCl), E6AP behaves as monomer (left panel). Molecular mass standards (right panel) were from the high molecular weight gel filtration calibration kit (GE Healthcare).

Supplementary Fig. 7

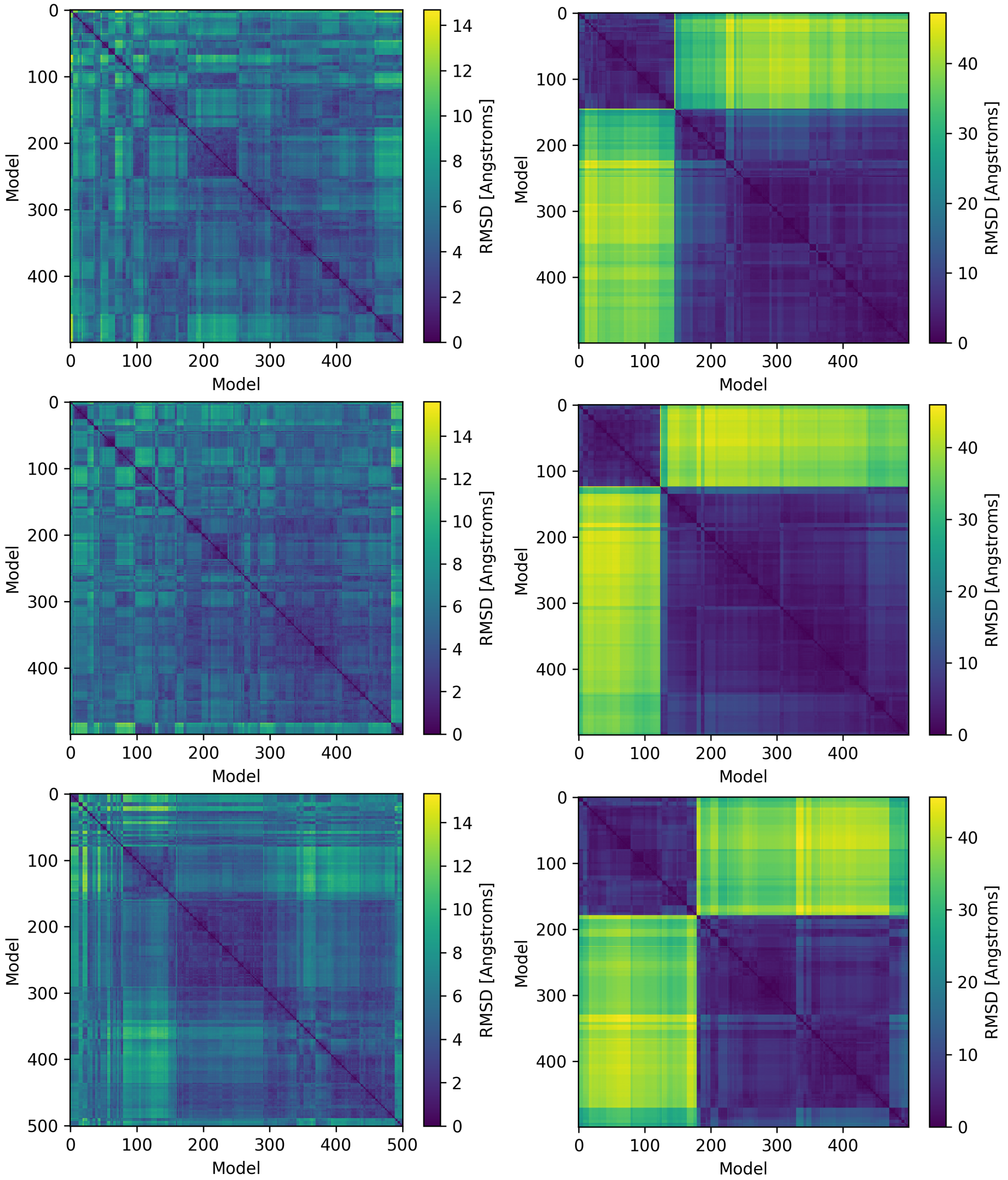

Supplementary Fig. 7 Matrix with best-scoring models.

Rmsd matrices of the 500 best-scoring models of the pooled sampling runs without (upper left) and with p53 (upper right). The run without p53 converged into a single cluster, while the run with p53 converged into one main cluster containing 341 members. The middle row shows the clusters for the jackknife runs (randomly removing 15% of our crosslink input database), and the lower row shows the runs containing only crosslinks with an ld-score greater than 30.

Supplementary Fig. 8

A B C D E F

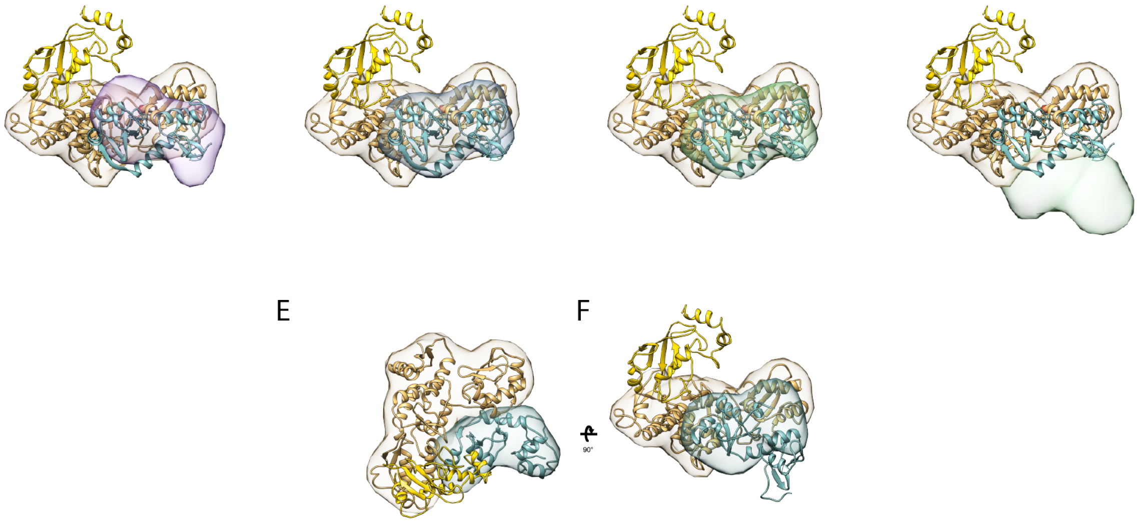

Supplementary Fig. 8 Structural models of the binding interface between the HECT domain of E6AP and E6 derived by an additional jackknifing approach, in which complete uxIDs were purposely removed, as well as by an independent modeling approach using HADDOCK confirm overall robustness and reproducibility of the modeling results.

Shown is the original structural alignment of the proteins including the center model of E6 overlaid with the density maps from the various modeling runs missing all crosslinks between E6AP_K708 – E6_K122 (violet) (A), E6AP_K779 – E6_K72 (B), E6AP_K799 – E6_K72 (C) or E6AP_K841– E6_K108 (D), respectively. Removing the *uxID* E6AP_K708 – E6_K122 leads to the overall same location of the density map with only a marginal shift in orientation. The run contains altogether five roughly equally populated clusters that are all centred around the same position and where the general orientation of the density maps remains the same, with only slightly different shifts in their respective angels. It can also be clearly seen from the models that the complete exclusion of two additional *uxID* restraints (E6AP_K779 – E6_K72 and E6AP_K799 – E6_K72) has virtually no influence on our models, whereas removal of all unique crosslinks for the uxID E6AP_K841– E6_K108 leads to a discernible shift in the respective density map. However, even in this model the main findings from our study – i.e. the binding site of E6 is in the vicinity of the catalytic centre of E6AP and distinct from the binding site of UbcH7, the cognate E2 of E6AP - is still valid.

We additionally used our crosslinking restraints as input for HADDOCK. (E) and (F) show the structural model of the binding interface between the HECT domain of E6AP and E6 generated by HADDOCK which we superimposed on the density map from the original structural model from our IMP based modeling runs by aligning the E6AP HECT domains from the HADDOCK and IMP models. It can clearly be seen that the model using HADDOCK generates a nearly identical binding interface between the HECT domain of E6AP and E6, where the E6 structure of the HADDOCK model fits almost completely into the E6 density map of the IMP model. In summary, both our additional modeling runs, where we performed a rigorous jackknifing approach, in which complete uxIDs were purposely removed, as well as an independent modeling approach using HADDOCK clearly confirm that our modeling results are highly robust and reproducible.

Supplementary Fig. 9

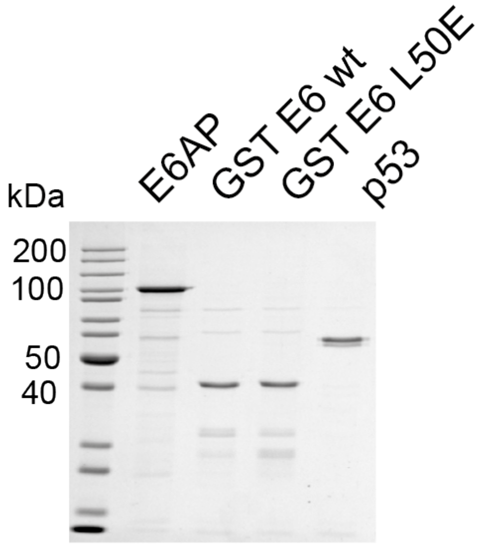

Supplementary Fig. 9 Proteins used for XL-MS experiments with p53.
SDS-polyacrylamide gel showing purified E6AP, wild-type E6 (note that a GST-E6 fusion protein was employed), the E6_L50E mutant (i.e. GST-E6 fusion protein), and p53 used for XL-MS experiments; 1 percent of the protein amount used for XL-MS was loaded. Proteins were visualized by Coomassie blue staining.
