# Supplementary material for: Structural dynamics of the E6AP/UBE3A-E6-p53 enzyme-substrate complex
Source: Nat Commun. 2018 Oct 25;9:4441. doi: 10.1038/s41467-018-06953-0 (PMC6202321; doi:10.1038/s41467-018-06953-0)
Supplement: Supplementary file 3 — Description of Additional Supplementary Files [file 41467_2018_6953_MOESM3_ESM.pdf]

Description of Additional Supplementary Files

Name: Supplementary Data 1

Description: XL-MS data for crosslinking of E6AP in the absence of E6

Column header	Description
Crosslinked peptide	Amino acid sequence of peptide a and peptide b of crosslinked peptide and position of linker attachment in peptide a and b.
Protein 1	Name of protein to which peptide a belongs to.
Protein 2	Name of protein to which peptide b belongs to.
Type	Type of linker attachment (inter-protein crosslink, intra-protein crosslink or monolink).
Pos 1	Position of lysine residue involved in crosslink formation in the amino acid sequence of protein 1. (Protein sequence without affinity tag for purification and in line with *UniProt* annotation was used).
Pos 2	Position of lysine residue involved in crosslink formation in the amino acid sequence of protein 2. (Protein sequence without affinity tag for purification and in line with *UniProt* annotation was used).
deltaS	Delta score is a measure for how close the best assigned hit scored in regard to the second best.
ID-Score	Linear discriminant score. Scoring scheme to discriminate between true-positive crosslinks and false positive hits.
Biological replicate	Indicates if the peptide was identified in biological replicate I, II or III.

Name: Supplementary Data 2

Description: XL-MS data for crosslinking of E6AP in presence of wild-type E6

Column labels are the same as described in the legend to Supplementary Data 1.

Name: Supplementary Data 3

Description: XL-MS data for crosslinking of E6AP in presence of the E6_L50E mutant

Column labels are the same as described in the legend to Supplementary Data 1.

Name: Supplementary Data 4

Description: Overview of identified crosslinked peptides

Column header	Description
Crosslinked peptide	Amino acid sequence of peptide a and peptide b of crosslinked peptide and position of linker attachment in peptide a and b.
Pos 1	Position of lysine residue involved in crosslink formation in the amino acid sequence of protein 1. (Protein sequence without affinity tag for purification and in line with *UniProt* annotation was used).
Pos 2	Position of lysine residue involved in crosslink formation in the amino acid sequence of protein 2. (Protein sequence without affinity tag for purification and in line with *UniProt* annotation was used).
Protein 1	Name of protein to which peptide a belongs to.
Protein 2	Name of protein to which peptide b belongs to.
Type	Type of linker attachment (inter-protein crosslink, intra-protein crosslink or monolink).
E6AP (Fig. 1A)	x indicates if peptide was identified in the experiment in which only the E6AP protein was crosslinked (see figure 1A).
E6AP + E6 (Fig. 1B)	x indicates if peptide was identified in the experiment in which E6AP protein was crosslinked in presence of E6 wild type protein (see figure 1B).
E6AP + E6 L50E mutant (Fig. 1C)	x indicates if peptide was identified in the experiment in which E6AP protein was crosslinked in presence of E6 L50E mutant protein (see figure 1C).
E6AP + E6 and p53	x indicates if peptide was identified in the experiment in which E6AP protein was crosslinked in presence of E6 wild type protein and p53 (see figure 4A).

Name: Supplementary Data 5

Description: Quantitative XL-MS for E6AP ± E6

Column header	Description
Protein	Protein to which quantified peptide belongs to.
uxID (pos1)	Position of lysine residue 1 of quantified unique crosslinking site (uxID). (Protein sequence without affinity tag for purification and in line with *UniProt* annotation was used).

uxID (pos2)	Position of lysine residue 2 of quantified unique crosslinking site (uxID). (Protein sequence without affinity tag for purification and in line with *UniProt* annotation was used).
Type	Type of linker attachment (inter-protein crosslink, intra-protein crosslink or monolink).
Fold change (log2 ratio)	Changes in abundance of the crosslinked peptides belonging to one uxID are expressed as $\log_2$ ratio (e.g. abundance with E6 / abundance without E6). In this study only changes that showed at least a change of log2ratio ≥ 1.5 (green) or log2ratio ≤ -1.5 (red) were considered significant changes.
pvalue	Indicates the regression between the two conditions.
sum_violations	Sum of violations recorded for each unique crosslinking site (uxID). For this study only uxIDs with 0 violations were considered.
imputed values	Unique crosslinking sites for which peptides were only identified in one of the two (or multiple) samples are assigned a fractional value in order to allow comparison also for crosslinks that are present in only one state: '==' indicates that a signal in both experiment and reference experiment was detected, '>=' indicates that only a signal in the experiment dataset and no signal in the reference experiment was detected and '<=' indicates that only a signal in the reference experiment could be detected.

Name: Supplementary Data 6

Description: Quantitative XL-MS for E6AP ± E6_L50E

Column labels are the same as described in the legend to Supplementary Data 5.

Name: Supplementary Data 7

Description: SILAC-XL-MS control data for crosslinking of light GST

Column labels are the same as described in the legend to Supplementary Data 1.

Name: Supplementary Data 8

Description: SILAC-XL-MS data for crosslinking of light and heavy labeled GST

Column labels are the same as described in the legend to Supplementary Data 1.

Name: Supplementary Data 9

Description: SILAC-XL-MS data for crosslinking of unlabeled (light) and isotope-labeled (heavy) E6AP in absence of E6

Column labels are the same as described in the legend to Supplementary Data 1.

Name: Supplementary Data 10

Description: SILAC-XL-MS data for crosslinking of unlabeled and isotope-labeled (heavy) E6AP in presence of E6

Column labels are the same as described in the legend to Supplementary Data 1.

Name: Supplementary Data 11

Description: XL-MS data for crosslinking of E6AP in presence of E6 and p53

Column labels are the same as described in the legend to Supplementary Data 1.

Name: Supplementary Data 12

Description: SILAC-XL-MS data for crosslinking of untreated light GST

Column labels are the same as described in the legend to Supplementary Data 1.

Name: Supplementary Data 13

Description: Extended data for quantitative XL-MS of E6AP ± E6 in Supplementary Table 5

**Column header**	**Description**
**a_dbID**	Identifier of the bagcontainer array in database.
**a_experimentname**	Experiment name is set during xTract analysis by the user in the experiment definition file. Here T1 = reference experiment.
**a_nbags**	Number of bags
**a_nlabes**	Number of labels
**a_uID**	Crosslink identifier: uID is defined by the sequence of the crosslinked peptides a and b, the position of the crosslinked lysine residues in peptide a and peptide b, the charge state of the crosslink, the type of the crosslink and the position and mass of a modification (if present).

**a_uxID**	Crosslink identifier: uxID is defined by the absolute positions of the crosslinked lysine residues in the respective protein sequence.
**b_heavy_mmax_area_sum_isotopes**	Mean of the maximum values of an isotope peak group for the heavy crosslink
**b_heavy_mmax_pg_tr_max**	Maximum retention time
**b_heavy_mmax_vTR**	Transformed retention time
**b_heavy_msum_area_sum_isotopes**	Mean of the summed areas of an isotope peak group for the heavy crosslink
**b_heavy_msum_pg_tr_max**	Maximum retention time
**b_heavy_msum_vTR**	Transformed retention time
**b_light_mmax_area_sum_isotopes**	Mean of the maximum values of an isotope peak group for the light crosslink
**b_light_mmax_pg_tr_max**	Maximum retention time
**b_light_mmax_vTR**	Transformed retention time
**b_light_msum_area_sum_isotopes**	Mean of the summed areas of an isotope peak group for the light crosslink
**b_light_msum_pg_tr_max**	Maximum retention time
**b_light_msum_vTR**	Transformed retention time
**c_violations**	Number of violations recorded

Name: Supplementary Data 14

Description: Extended data for quantitative XL-MS of E6AP ± E6 in Supplementary Table 5

**Column header**	**Description**
**a_bag_container_compare_labels_log2ratio**	Log2ratio calculated during compare labels validation. The mean of the sums or the mean of the maximum values are compared as defined by user. A violation is recorded if the differences are larger than the threshold value.
**a_bag_container_db_index**	Identifier of the bagcontainerarray in database.
**a_bag_container_violations**	Number of violations at the bagcontainer level.
**a_bag_db_index**	Identifier of the bag in database.
**a_bag_n_violations**	Number of violations at the peptide bag level.

**a_peptide_db_index**	Identifier of the peptide in database.
**b_peptide_decoy**	Target IDs: parameter is set to 0; Decoy IDs: parameter is set to 1.
**b_peptide_fdr**	False discovery rate (FDR) of peptide (calculated during xProphet analysis).
**b_peptide_idx**	Identifier of peptide.
**b_peptide_mod**	Position and mass of a peptide modification (if present).
**b_peptide_ms2evidence**	For peptides that have a MS2 evidence this parameter is set to 1. If the peptide has no MS2 evidence in that run the parameter is set to 0.
**b_peptide_msrunbase**	Indicates to which measurement the data belongs (name of profile file).
**b_peptide_mz**	Mass to charge ratio of precursor ion.
**b_peptide_nIDs**	Number of IDs.
**b_peptide_npgs**	Number of peak groups.
**b_peptide_preINT**	Intensity of precursor ion.
**b_peptide_prot**	Protein to which peptide a (and peptide b) of crosslinked peptide belong to.
**b_peptide_scan**	MS scan in which peptide was identified (only valid if b_peptide_ms2evidence = 1).
**b_peptide_score**	Score of peptide calculated during xQuest analysis.
**b_peptide_seq**	Amino acid sequence of peptide.
**b_peptide_tr**	Retention time of peptide.
**b_peptide_transition_group_id**	Identifier for the peptide transition group.
**b_peptide_type**	Type of peptide (xlink or monolink; light or heavy)
**b_peptide_uID**	uID of the peptide. uID is defined by the sequence of the crosslinked peptides a and b, the position of the crosslinked lysine residues in peptide a and b, the charge state of the crosslink, the type of the crosslink and the position and mass of a modification (if present).
**b_peptide_uxID**	uxID of the peptide: uxID is defined by the absolute positions of the crosslinked lysine residues in the respective protein sequence.

**b_peptide_var_valid**	The variable 'valid' is set to 0 if the xtracted peptide has no peak group defined.
**b_peptide_z**	Charge state of crosslinked peptide.
**c_pg_LD1**	LD1 of the respective peak group
**c_pg_area_sum_isotopes**	The areas of the individual isotopes are calculated and individual areas of the isotopes belonging to one peak group are summed.
**c_pg_area_sum_isotopes_org**	Original value of summed areas.
**c_pg_c_pg_pgid**	Peak group ID.
**c_pg_d_score**	Normalized discriminant score calculated by mProphet. Used to rank the peak groups.
**c_pg_decoy**	Indicates if peak group is a decoy hit.
**c_pg_deltatr_sec**	Retention time shift in seconds. Delta tr of the maximum of the isotopes compared to the maximum of the summed scans is calculated.
**c_pg_dynamicrangefilter**	Dynamic range filter used to filter out peak groups.
**c_pg_idx**	Index of peak group.
**c_pg_m_score**	mProphet score (m_score). Indicates the identification confidence of the corresponding peak group.
**c_pg_main_var_sumscore**	Statistical value.
**c_pg_max_int_first_iso**	Maximum intensity of the first isotope (monoisotopic peak).
**c_pg_max_int_first_iso_org**	Original value of maximum intensity.
**c_pg_max_int_iso**	Maximum intensity of the isotopes.
**c_pg_mean_delta_TR_all_IDs**	Mean distance of the peptide-spectrum matches (PSMs) in a peak group relative to the peak group apex.
**c_pg_ms2evidence**	Indicates if there was a MS2 evidence and a identification for the corresponding peak group; yes=1 ; no=0
**c_pg_msrunbase**	Indicates to which measurement the data belongs (name of profile file).
**c_pg_nIDs**	Number of IDs that were used to extract.
**c_pg_nMS2**	Number of IDs that match the peak group (total).
**c_pg_nMS2e**	Number of IDs that match the peak group + tolerance and are from this MS run.

**c_pg_npgs**	Number of matched PSMs in each peak group.
**c_pg_peak_group_rank**	Peak group rank.
**c_pg_pg_tr_max**	Maximum retention time of the peak group.
**c_pg_pg_tr_width**	Retention time width of the peak group.
**c_pg_pgid**	Peak group identifier
**c_pg_stdev_delta_TR_all_IDs**	Standard deviation of distance of the peptide-spectrum matches (PSMs) in a peak group relative to the peak group apex.
**c_pg_sum_delta_tr_iso**	Sum of retention-time deviation of isotopes belonging to one peak group.
**c_pg_transition_group_id**	Unique identifier of the extraction.
**c_pg_transition_group_record**	Unique identifier for a single measurement of a transition group.
**c_pg_uID**	uID of the peak group. uID is defined by the sequence of the crosslinked peptides a and b, the position of the crosslinked lysine residues in peptide a and b, the charge state of the crosslink, the type of the crosslink and the position and mass of a modification (if present).
**c_pg_vTR**	Retention time transformed to vconsensusrun.
**c_pg_var_P_binom_nMS2**	MatchOdds-based score. This score uses a binominal distribution to calculate the probability that a certain number of PSMs matches to a given peak group. The prior probability for matching is defined by the width oft he peak group (including tolerance for matching) and the queried retention-time space. The score is then transformed to a scale of 0-1, where a higher score reflects the case where the observed count is unlikely to be observed by chance.
**c_pg_var_cdf_delta_TR_all_IDs**	Retention-time deviation score. Probability-based score using a (Gaussian) cumulative distribution function (CDF). Calculates the mean distance of the peptide-spectrum matches (PSMs) in a peak group relative to the peak group apex.
**c_pg_var_cdf_deltratr**	Retention-time deviation score. Probability-based score using a (Gaussian) cumulative distribution function (CDF). Defined by the distance of the peak group apex to the expected retention time.
**c_pg_var_corr**	Correletion score which is calculated on the basis of the comparison of theoretical and observed isotope intensities. Corresponds to Pearson correlation coefficient (PCC).

**c_pg_var_nmsc**	Normalized spectral angle score which is calculated on the basis of the comparison of theoretical and observed isotope intensities. Corresponds to the normalized spectral contrast angle.
**d_exp_biol_rep**	Number of biological replicate. Specified by user in experiment definition file.
**d_exp_fraction**	Number of fraction. Specified by user in experment definition file.
**d_exp_name**	Experiment name is specified during xTract analysis by the user in the experiment definition file. Here T1 = reference experiment.
**d_exp_tech_rep**	Number of technicel replicate. Specified by user in experiment definition file.

Name: Supplementary Data 15

Description: Extended data for quantitative XL-MS of E6AP ± E6_L50E in Supplementary Table 6

Column labels are the same as described in the legend to Supplementary Data 13.

Name: Supplementary Data 16

Description: Extended data for quantitative XL-MS of E6AP ± E6_L50E in Supplementary Table 6

Column labels are the same as described in the legend to Supplementary Data 14.
